# Unravelling the tumour genome: The evolutionary and clinical impacts of structural variants in tumourigenesis

**DOI:** 10.1002/path.5901

**Published:** 2022-04-28

**Authors:** Alhafidz Hamdan, Ailith Ewing

**Affiliations:** ^1^ MRC Human Genetics Unit, Institute of Genetics and Cancer University of Edinburgh Edinburgh UK; ^2^ Cancer Research UK Edinburgh Centre, Institute of Genetics and Cancer University of Edinburgh Edinburgh UK

**Keywords:** structural variants, whole genome sequencing, tumour evolution, chromothripsis, extrachromosomal DNA, patient stratification

## Abstract

Structural variants (SVs) represent a major source of aberration in tumour genomes. Given the diversity in the size and type of SVs present in tumours, the accurate detection and interpretation of SVs in tumours is challenging. New classes of complex structural events in tumours are discovered frequently, and the definitions of the genomic consequences of complex events are constantly being refined. Detailed analyses of short‐read whole‐genome sequencing (WGS) data from large tumour cohorts facilitate the interrogation of SVs at orders of magnitude greater scale and depth. However, the inherent technical limitations of short‐read WGS prevent us from accurately detecting and investigating the impact of all the SVs present in tumours. The expanded use of long‐read WGS will be critical for improving the accuracy of SV detection, and in fully resolving complex SV events, both of which are crucial for determining the impact of SVs on tumour progression and clinical outcome. Despite the present limitations, we demonstrate that SVs play an important role in tumourigenesis. In particular, SVs contribute significantly to late‐stage tumour development and to intratumoural heterogeneity. The evolutionary trajectories of SVs represent a window into the clonal dynamics in tumours, a comprehensive understanding of which will be vital for influencing patient outcomes in the future. Recent findings have highlighted many clinical applications of SVs in cancer, from early detection to biomarkers for treatment response and prognosis. As the methods to detect and interpret SVs improve, elucidating the full breadth of the complex SV landscape and determining how these events modulate tumour evolution will improve our understanding of cancer biology and our ability to capitalise on the utility of SVs in the clinical management of cancer patients. © 2022 The Authors. *The Journal of Pathology* published by John Wiley & Sons Ltd on behalf of The Pathological Society of Great Britain and Ireland.

## Introduction

During their evolution from normal cells to invasive tumours, cells acquire somatic mutations throughout their entire genome. Structural variants (SVs) represent an underappreciated class of genomic aberration that may amplify, delete, and rearrange genomic regions either focally as a simple change to a single genomic segment, or catastrophically, combining multiple SVs, encompassing large genomic regions and involving multiple chromosomes. Tumours harbour a diverse range of structural events along this spectrum of increasing size and complexity. While copy number alteration (CNA) and aneuploidy have been well studied in tumours [1, 2], the impacts that more complex classes of SV have on tumour development and progression are less well understood. However, the increased availability of whole‐genome sequencing (WGS) data from tumours generated by large‐scale studies such as The Cancer Genome Atlas (TCGA) [3], the Pan‐Cancer Analysis Group of the International Cancer Genome Consortium (PCAWG‐ICGC) [4, 5, 6] and the Hartwig Medical Foundation (HMF) [7], combined with advances in the methods required to interrogate SVs, allow us to examine SVs in tumours at increased depth and scale. Nevertheless, our understanding of the critical role that SVs have in tumourigenesis has been slower to emerge as accurate and comprehensive detection of SVs is challenging due to the diversity in types, sizes, and complexity of SVs in tumours. Some SVs can drive tumourigenesis, while others shed light on the processes of mutation and repair acting during tumour development [8]. Evolutionary histories of cancer genomes reveal the major role of SVs in tumour evolution across many cancer types and the potential of SVs as biomarkers to inform the treatment of cancer patients at various stages of their disease is emerging. In this review, we discuss recent challenges and developments in detecting SVs, present the important roles of SVs in cancer progression and in generating intratumoural heterogeneity, and highlight the emerging clinical applications of SVs as biomarkers in cancer.

## The structural complexity of tumour genomes

**Figure 1:**
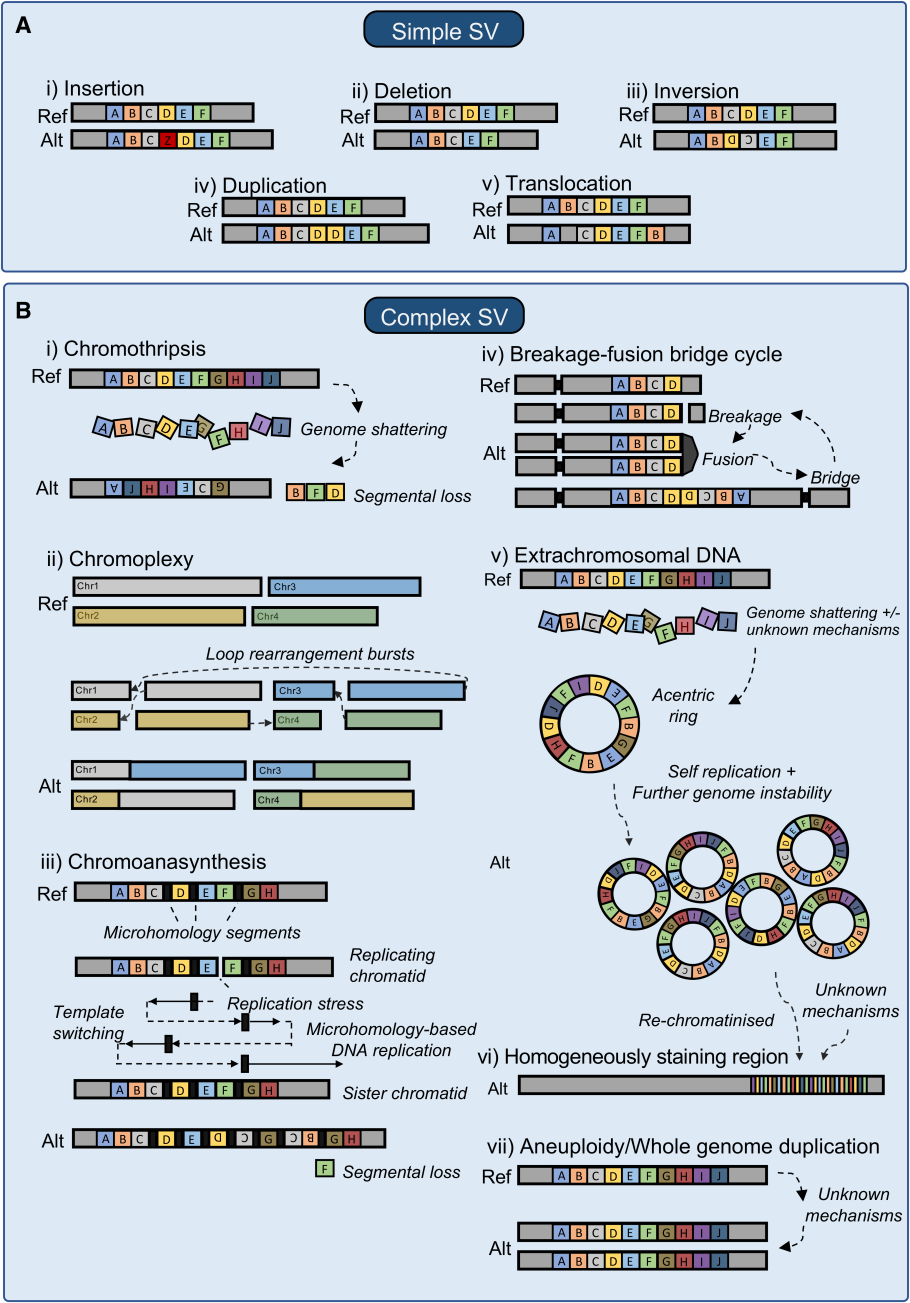
The diverse structural variant (SV) landscape in tumours. (A) Simple SVs include (i) insertions where segments of DNA are added, (ii) deletions where DNA segments are lost, (iii) inversions where DNA segments are in the opposite orientation, (iv) duplications where extra copies of DNA segments are present, and (v) translocations where a section of DNA is joined onto another section at a different genomic location. (B) Complex SVs include: (i) chromothripsis where the genome is shattered and the pieces joined back together at random, (ii) chromoplexy where interdependent translocations and deletions are joined together in a chain, (iii) chromoanasynthesis, which is a pattern of localised amplification and short stretches of breakpoint microhomology, (iv) breakage‐fusion‐bridge cycles, where telomeres are lost, dicentric chromosomes form before breaking due to mitotic stress, (v) extrachromosomal DNAs (ecDNAs), where large segments of DNA form acentric circles that often contain oncogenes and regulatory regions; these ecDNAs may be reincorporated into the chromosome, as (vi) homogeneously staining regions, and (vii) aneuploidy and whole‐genome duplication where some or all chromosomes have additional or double the number of copies, respectively.

SVs are frequently found in the normal human genome and contribute greatly to individual human variation [9, 10, 11]. What differentiates tumour genomes from normal genomes are the unusual clusters, types, and sizes of SVs, and in highly rearranged tumours, the increased frequency and complexity of SVs relative to that observed in normal tissues [5]. SVs are formed by the improper repair of double‐stranded DNA breaks [12, 13, 14]. These DNA breaks are triggered by various mechanisms such as exogenous mutagens (DNA damaging chemicals, viral infections, ionising radiation, and ultraviolet light) and cell‐intrinsic mutational processes (oxidative metabolism, telomere dysfunction, and replication stress); in‐depth reviews of the mechanisms contributing to SV formation are detailed elsewhere [13, 14, 15, 16]. SVs can reconfigure DNA sequences across scales from a single genomic locus to multiple entire chromosomes. Simple SVs are single‐segment alterations that include insertions, deletions, duplications, inversions, and translocations (Figure 1A). Complex SVs constitute multiple simple SVs whose co‐occurrence in a genome, within a spatial and/or temporal window, represent the expected genomic consequences of complex events. These consequences, in the form of clusters of SVs, are used to predict the past occurrence of a diverse set of complex events including chromothripsis, chromoplexy, chromoanasynthesis, breakage‐fusion‐bridge (BFB) cycles, extrachromosomal circular DNA (ecDNA), aneuploidy, and whole‐genome duplication (WGD) (Figure 1B). Briefly, chromothripsis is defined by extensive genomic rearrangement localised to a single or sometimes a few chromosomes. The hallmarks of chromothripsis include oscillations between two or three copy number states, random reassembly of fragmented DNA segments, and loss of heterozygosity [17, 18]. Chromoplexy contains interdependent translocations and deletions of multiple chromosomal segments organised in a closed chain [19]. Chromoanasynthesis involves serial microhomology‐based breakage‐induced replication or fork stalling and template switching mechanisms, resulting in localised duplications and triplications and short stretches of microhomologies at breakpoint junctions [20]. BFB cycles occur as a result of telomere loss, dicentric chromosome formation, and mitotic spindle stress [21, 22]. ecDNAs are large, typically more than 1 Mb in length, acentric circular DNAs that are independent from chromosomes and often contain oncogenes and regulatory regions [23, 24]. ecDNAs have previously been described as ‘double minutes’ due to their appearance as paired structures [25]. These ecDNA segments can be reincorporated into the chromosome as large sections of tandem duplications known as homogeneously staining regions (HSRs) [26]. Aneuploidy is the presence of an abnormal number of chromosomes [27], whereas WGD results from the doubling of a complete set of diploid chromosomes [28].

Despite the wide variety in the complex classes of SVs that have been defined, recent large‐scale studies have identified a large fraction of clustered SVs, which are not thought to be the consequence of a particular known complex event as defined in Figure 1B [5, 29]. These unclassified SV clusters represent a gap in our understanding of the highly rearranged tumour genome. It is unclear what proportion of these clusters represent as‐yet uncharacterised classes of complex events or whether they remain unclassified due to significant flexibility in the current genomic definitions of known complex events. For example, arbitrary thresholds are often applied in defining the genomic consequences of complex events such as chromothripsis [18], and complex SVs that do not meet the arbitrary thresholds will not be classified as such. With the increasing availability of long‐read WGS data and improvements in methods for SV detection and analysis, we expect that more classes of complex events will be uncovered and that definitions of the genomic consequences of complex events will be refined. A more recent study [30], using a new algorithm to classify SVs, identified several novel classes of complex SV, the mechanistic origins of which have yet to be explored [31]. These include rigma (large deletions at fragile sites), pyrgo (superenhancer associated duplicated regions) and tyfonas (amplified areas of fold‐back inversions). Further, tracing the sequential formation of complex SVs reveals more complex patterns of rearrangements, such as the recently reported ‘seismic amplifications’ [32]: amplifications which are thought to occur as a result of circular recombination of chromothripsis‐generated ecDNAs. These wave‐like patterns of amplification can be integrated into the chromosome as HSRs or may persist as ecDNAs [33]. Although complex SVs represent a variety of intriguing genomic alterations, this heterogeneous class of mutation is still to be comprehensively characterised, as reflected in the dynamic and often overlapping definitions of these variants. The ability to generate accurate observations of the structural complexity of tumour genomes will benefit the comprehensive analysis of large tumour cohorts and experimental studies of the mechanisms underlying complex events.

## New horizons in SV detection

Substantial diversity in the type, size, and complexity of SVs in tumours poses a challenge for accurate detection. Historically, SVs were detected by microscopic karyotyping, fluorescence *in situ* hybridisation (FISH), and microarray technologies; providing limited SV breakpoint resolution and obscuring complex events. In the last two decades, short‐read WGS has revolutionised SV detection, providing both higher accuracy and resolution; and much of our current knowledge on the diversity of SVs in cancer is derived from short‐read WGS data. In‐depth reviews on short‐read WGS‐based SV detection are discussed elsewhere [8, 34, 35, 36]. Briefly, nucleic acids extracted from tumour tissues and a germline control (usually a blood sample) are PCR (polymerase chain reaction)‐amplified, sequenced as paired‐end reads of 100–300 base pairs in length, and computationally aligned to the reference genome. SV detectionalgorithms (callers) are then used to identify the SVs unique to the tumour sample. Many SV callers have been developed, and their relative merits have been reviewed in detail [8, 37, 38]. SV callers leverage distinct alignment signatures to identify SVs by type and size [39]. Changes in read depth are used together with allelic imbalances to infer CNAs [40, 41, 42]. Clusters of discordantly aligned read pairs (reads that map at abnormal distance or orientation) together with split reads (reads that are partially mapped) can provide precise SV breakpoint locations [43, 44, 45, 46, 47]. SVs can also be detected using a local assembly approach where aberrant reads are reassembled into contigs, before pairwise comparison to the reference genome [46, 47, 48]. Newer SV callers such as GRIDDS‐PURPLE‐LINX [49] and JaBbA [30] incorporate both SV and CNA information to reduce false‐positives and detect the presence of complex SVs. Specialised SV callers have also been developed, tailored to detect specific complex SVs, including ShatterSeek [18] (for chromothripsis) and Amplicon Architect [50] (for ecDNAs).

Irrespective of the strategies used to call SVs, no single short‐read WGS‐based SV caller can identify the complete range of SV types reliably, due to the structural complexity of tumour genomes and the technical limitations of short‐read lengths [51]. Despite our best efforts at optimising SV detection, a large swathe (~15%) of the genome remains inaccessible to short‐read WGS [52]. These genomic regions frequently harbour highly repetitive sequences or extreme GC content [10, 53]. PCR amplification introduces coverage bias across regions with extreme GC content [54]; this bias leads to the artificial reduction of supporting reads that can affect methods used to detect CNAs [40, 41, 42]. In addition, short‐read lengths are not able to span large regions of repetitive sequence in the genome, which are particularly prone to SV formation [14, 52]. Repetitive sequence causes increased alignment ambiguity particularly for short sequencing reads, reducing variant calling accuracy. Similarly, short‐read WGS cannot span most complex SVs in their entirety, leading to the incomplete classification of patterns of single SVs into complex SVs. Due to the read length limitation, short‐read WGS also cannot adequately phase complex SVs into their constituent haplotypes, thereby affecting the ability of short‐read WGS in resolving complex events at allelic resolutions [55]. These challenges to comprehensively detecting SVs, and mapping complex SVs from short‐read WGS suggest that we will have missed many SVs present in tumour genomes and may have underestimated the importance of SVs in tumour progression.

To overcome the limitations of short‐read WGS in fully resolving the structural complexity of tumour genomes, several approaches incorporating longer‐range information have been developed [56, 57, 58]. These include barcoding collections of short reads from the same genomic region to improve assembly accuracy [59, 60, 61, 62, 63, 64, 65, 66, 67, 68], and using fluorescently labelled sequence motifs to image very long contiguous stretches of DNA, termed optical mapping [69]. The former is still subject to the limitations of short‐read lengths and the latter, while useful in identifying complex SVs such as ecDNAs [50, 70, 71], is unable to identify precise breakpoint locations at high resolution. Alternatively, long‐read sequencing approaches, such as those developed by Oxford Nanopore Technologies (ONT) and Pacific Biosciences (PacBio) [58] can generate individual reads that are kilobases to megabases in length directly from the native DNA, often spanning SVs in their entirety. In‐depth reviews of the technologies underlying ONT and PacBio long‐read sequencing platforms are detailed elsewhere [56, 57, 58]. Both ONT and PacBio reads can readily traverse the most repetitive regions of the human genome, and lack GC bias, allowing for more comprehensive identification of SVs, including those that span repeat‐rich centromeric and telomeric regions [72, 73, 74, 75, 76, 77, 78]. Most of the SVs discovered using ONT and PacBio reads are novel, lending weight to the notion that a large number of SVs are not detected by short‐read approaches [52, 79, 80, 81]. In addition, longer read lengths allow haplotype phasing and *de novo* assembly of complex SVs, crucial for the accurate interpretation of the functional impact of these events [82, 83, 84, 85]. Taking advantage of the unique properties of both ONT and PacBio long‐read sequencing, and the development of new whole‐genome assembly methods [78, 86], the Telomere‐to‐Telomere Consortium has recently assembled a complete human genome for the first time [72, 87, 88]. In the future, we expect that this approach will be applied at scale to accurately resolve large complex SV events. With further refinement in long‐read sequencing technologies, including improvements in cost and throughput, and the development of more accurate SV detection algorithms [78, 89, 90], it is likely that long reads will be used more broadly in the future. Large scale initiatives involving the use of long‐read sequencing in cohorts with detailed clinical follow up data, such as Cancer 2.0 by Genomics England [91], represent a valuable opportunity to further our understanding of the full diversity and complexity of SVs and better evaluate their impact on tumour development and progression.

## Structural variation as a critical dimension in tumour evolution

Cancers evolve from single cells into genetically distinct and heterogeneous subpopulations of cells. Cells that acquire mutations that confer selective advantages undergo clonal expansion [92, 93]. The dynamics of somatic evolution are thus determined by the rate of mutation and the likelihood that a mutation confers a selective advantage [94]. High rates of mutation provide more opportunity for clonal expansion, while conversely increasing the likelihood of a clone acquiring a deleterious variant. Intratumour heterogeneity, during selective sweeps and in periods of neutral evolution where no cell subpopulation has a fitness advantage over another, offers a reservoir of mutations upon which selection could act in the presence of selective pressures; for example, as a result of therapy [92], driving malignant progression, and therapeutic resistance. Driver mutations can occur early as tumour‐initiating events or present late, contributing to tumour progression and metastasis. In a given tumour sample, these driver mutations might have propagated clonally and be present in all cells or might be subclonal, existing only in a subset.

The importance of SVs, in particular for the continued development of the tumour after the last clonal expansion, may be underappreciated due to the substantial technical challenges in identifying subclonal SVs [91]. Recent large‐scale studies suggest that SVs play a significant role in driving tumourigenesis, with more than 50% of cancers harbouring at least one clonal SV driver [91]. Additionally, only 11% of subclones carry a single nucleotide variant (SNV) or small indel driver, suggesting that late tumour development may be disproportionately driven by subclonal large CNAs or SVs [95]. Our ability to detect subclonal SVs is dependent on sequencing depth and quality, tumour cellularity, and background ploidy. The low allelic frequencies of subclonal SVs can present a substantial challenge for both accurate detection and probabilistic assignment of breakpoints to subclones using bulk sequencing methods, where our ability to detect SVs is impaired by sequencing a pooled sample from a heterogeneous population [96]. Single‐cell WGS has greatly helped to accurately determine the clonality of SVs [97, 98, 99, 100]. Regardless, challenges in establishing clonality will inevitably result in a substantial underestimation of the proportion of SVs that are subclonal. Nonetheless, based on current estimates, *when* SVs occur during tumour evolution shows striking differences across tumour types [101, 102]. For example, pilocytic astrocytomas and non‐Hodgkin lymphomas harbour predominantly clonal SV drivers which are likely to have occurred earlier in tumourigenesis, whereas leiomyosarcomas and ovarian adenocarcinomas contain frequent subclonal SV drivers, which are more likely to contribute to later development [95]. In glioblastomas and medulloblastomas, a substantial fraction of chromosomal gains occur very early in molecular time (within the first 10%), whereas in melanomas, lung cancers, and papillary kidney cancers, chromosomal gains occur late and towards the end of molecular time [101]. Further, driver clonality may depend on the type of SV. For example, there is some suggestion from their predominantly clonal presence in tumour samples that gain‐of‐function SVs, including certain recurrent oncogenic fusions such as *TMPRSS2‐ESG* and *BRAF‐KIAA1549*, occur early in tumour development [95].

Genomic instability is a feature of almost all human cancers and allows tumour cells to develop the capabilities required to survive, proliferate, and spread [103, 104, 105, 106]. Conceptually, genomic instability represents an increased tendency for genomic alteration in tumour cells during cell division, occurring as a result of defective surveillance mechanisms governing genomic integrity [107]. Historically, genomic instability in cancer genomes has been thought to follow a stepwise process in which drivers accumulate gradually over time [93, 108, 109]. This is in contrast to the ‘Big Bang’ model of evolution which posits that tumours grow as a single terminal expansion, producing heterogeneous subclones at tumour initiation [110, 111]. In this model, the driver alterations required for tumour initiation are present early and are sufficient to support subsequent expansion [110]. In most tumour types there is a wide temporal window of genomic instability; however, this is not always the case [101]. Recent large‐scale studies suggest that complex SVs such as chromothripsis [17] and chromoplexy [19, 112, 113] typically occur within a single event [17]. Such mutational bursts suggest that cancer cells can alternate long phases of latency with short periods of intense, punctuated events [109, 112, 114], allowing cancer cells to attain greater fitness than would be possible through a gradual or stepwise accumulation of alterations [18]. Within a cancer genome, complex events can occur sequentially, suggesting that they may be mechanistically interlinked, in that the occurrence of one event increases the likelihood of a second. For example, dicentric chromosomes generated by BFB cycles and telomere attrition are precursors to chromothripsis [115, 116]. Similarly, the formation of a complex SV may facilitate the development of another one. For example, chromothripsis frequently occurs after the onset of WGD, with the latter presumably providing cells with sufficient intact genetic material to withstand the onslaught of genomic instability of the former [117]. In support of this, WGD commonly occurs in the intermediate stages of evolution in various cancers [101, 118].

Another form of accelerated evolution is mediated by ecDNAs. Because ecDNA fragments lack centromeres, the molecules are not attached to the mitotic spindle during cell division, leading to uneven segregation into daughter cells [119, 120, 121]. The resultant stochastic non‐Mendelian inheritance leads to rapid and dynamic changes of oncogene content under selection pressure, in response to tumour microenvironment and targeted therapies, leading to treatment resistance [122, 123, 124, 125]. In addition, circular recombination can occur after ecDNA formation, as a result of ongoing mutational processes [32]. In this instance, rapid alterations of the DNA sequence within the ecDNA can occur outside of cell division *via* complex events such as chromothripsis, providing another dimension to the evolution of structural complexity in the tumour genome [33]. SVs, and particularly complex SVs, play a significant yet underappreciated role in determining how cancer genomes evolve. Single‐cell WGS applied to organoid systems [126, 127] has enabled high‐resolution phylogenetic studies and functional interrogation of subclonal SVs [128]. In addition, longitudinal single‐cell WGS of patient‐derived xenografts from tumour samples has demonstrated that CNAs have an underappreciated impact on clonal fitness [129]. The evolutionary trajectories of structural complexity in tumours significantly impact how tumours progress but vary substantially between tumours and particularly between cancer types. The potential utility of these evolutionary trajectories in informing our understanding of tumour biology and as clinical biomarkers for treatment response and disease progression represents a currently untapped translational opportunity.

## The functional impacts of structural complexity

**Figure 2:**
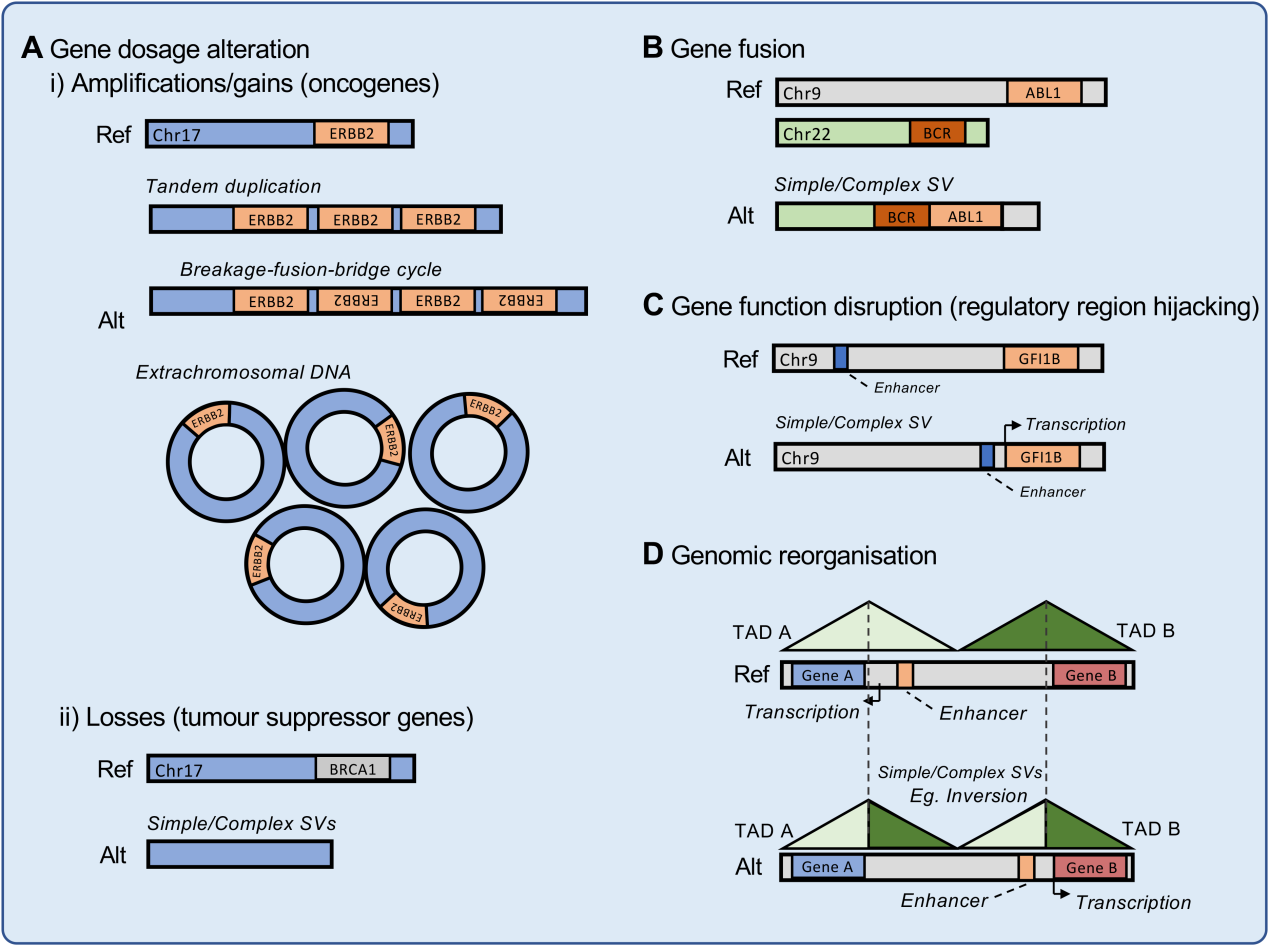
Pathogenic consequences of structural variants (SV). (A) Gene dosage alteration: SVs may (i) increase the expression of an oncogene by having extra copies as a result of amplifications/gains, mediated by various mechanisms such as tandem duplication, breakage‐fusion‐bridge cycle, and extrachromosomal DNA, or (ii) decrease the expression of tumour suppressor genes by reducing their copy number. (B) Gene fusion: translocations may bring two genes together to form a novel gene fusion, which may result in the production of novel fusion proteins beneficial to the cancer. (C) Gene function disruption: SVs may disrupt the function of genes *via* regulatory hijacking, for example by bringing an enhancer closer to a proto‐oncogene, thereby increasing its expression. (D) Genomic reorganisation: SVs may rearrange the wider chromosomal architecture, for example by rearranging the boundaries of topological associated domains, potentially bringing closer ectopic enhancers that would activate proto‐oncogenes, increasing their expression.

Recent studies indicate that pathogenic SVs occur in at least 30% of all cancers [8, 101, 130]. SVs that are pathogenic disrupt the function of oncogenes or tumour suppressor genes, by increasing or decreasing their expression, respectively. The most well‐studied pathogenic consequences of SVs are their direct impacts on genes, either *via* gene dosage alteration (Figure 2A) or by the creation of gene fusions (Figure 2B). Gene dosage alterations occur *via* unbalanced rearrangements from simple SVs such as deletions and duplications, which may encompass entire chromosomes (aneuploidy) or even genomes (WGD). Alternatively, complex SVs such as chromothripsis and ecDNAs generate CNAs [101, 131, 132, 133]. Gene fusions occur *via* rearrangements that cause juxtaposition of two genes normally at distant loci [112, 134, 135]. A canonical example of a pathogenic oncogenic fusion is the *BCR‐ABL1* oncoprotein, highly prevalent in chronic myeloid leukaemia [136, 137]. The functional and clinical impacts of simple CNAs, aneuploidies and fusion genes have been demonstrated across many cancers [138, 139], and form the basis for several highly effective clinical interventions described in the next section. However, WGS has enabled investigation of these events at a much greater depth and scale than was possible before and has highlighted alternative routes to gene dosage alteration that were previously obscured. SVs that occur within noncoding regulatory regions can cause indirect disruption of gene function. For example, in medulloblastoma, different SV classes juxtapose the proto‐oncogenes *GFI1* and *GFI1B* to distal active enhancer elements [140], increasing their expression. This phenomenon has been termed ‘enhancer hijacking’ (Figure 2C). Further, SVs can disrupt the function of oncogenes and tumour suppressor genes *via* alteration of higher‐order chromatin structure [141, 142, 143] (Figure 2D). A recent study by the PCAWG demonstrated that SVs affect topologically associating domain (TAD) boundaries in a cancer‐specific manner (according to overall SV burden), are able to generate new TAD structures, and can lead to marked changes in chromatin folding [142]. SVs often overlap with binding sites for the transcriptional regulator, CTCF, near proto‐oncogenes of certain cancer types, potentially resulting in disruption of CTCF‐CTCF chromatin folding loops [142]. Both disruption to TAD structures and CTCF‐CTCF chromatin folding loops result in genome restructuring, bringing ectopic enhancers close to proto‐oncogenes, leading to increased expression. Also, different classes of SVs may employ different mechanisms that converge on the same functional outcome, for example, altered gene expression, and these may occur independently or together within the same cancer genome. For example, in glioblastoma, the receptor tyrosine kinase signalling pathway can be altered by disruption of the *EGFR* oncogene, either *via* gene fusion (*EGFRvIII* fusion [144]) or gene dosage increase (*EGFR* amplification [145, 146]), caused by either simple or complex SVs [147, 148].

Assigning pathogenicity to a given SV can be challenging, as most SV breakpoints reside within the noncoding region of the genome [149], with no direct impact on the sequences of the coding genome. SVs such as inversions and complex SVs can have unpredictable functional impact, suggesting the presence of complex regulatory effects impacting long range noncoding regulatory elements discussed above rather than simple dosage alteration [150]. In addition, as a single SV commonly spans multiple genes, assigning pathogenicity to disruption of a particular gene can be difficult. Mutations driving tumour progression are usually recurrent, meaning that the same alteration results in the same phenotype across multiple cancer genomes or patients [151, 152]. Given the diversity in the types and sizes of SVs, the discovery of a recurrent distinct SV event is rare, making proving their role in driving tumourigenesis challenging. Furthermore, the combinatorial impacts of multiple SVs can be hard to resolve as phasing SVs, to ensure that they are impacting the same allele, based on short‐read WGS, is almost always impossible. These barriers to determining whether an SV is pathogenic lead to difficulties in determining the clinical impact of such events on patients. Nevertheless, a clinically significant SV does not necessarily have to be a driver of tumourigenesis. SVs can also be the consequence of a particular mutational processes or disrupted DNA repair mechanisms and can reveal insights into the presence or absence of these processes in a given tumour [153, 154]; these genome‐wide patterns of SVs can be clinically useful as biomarkers for treatment response. Lastly, SVs do not always promote tumour development, and in fact can be deleterious to the tumour. For example, deletions of *BRCA1/2* leads to DNA damage repair deficiencies in certain tumours [155, 156], and tumours with a large fraction of complex SVs generate vast amounts of neoantigens that may confer susceptibility to immune therapies [157, 158].

## The emerging clinical applications of SVs in cancer

**Figure 3:**
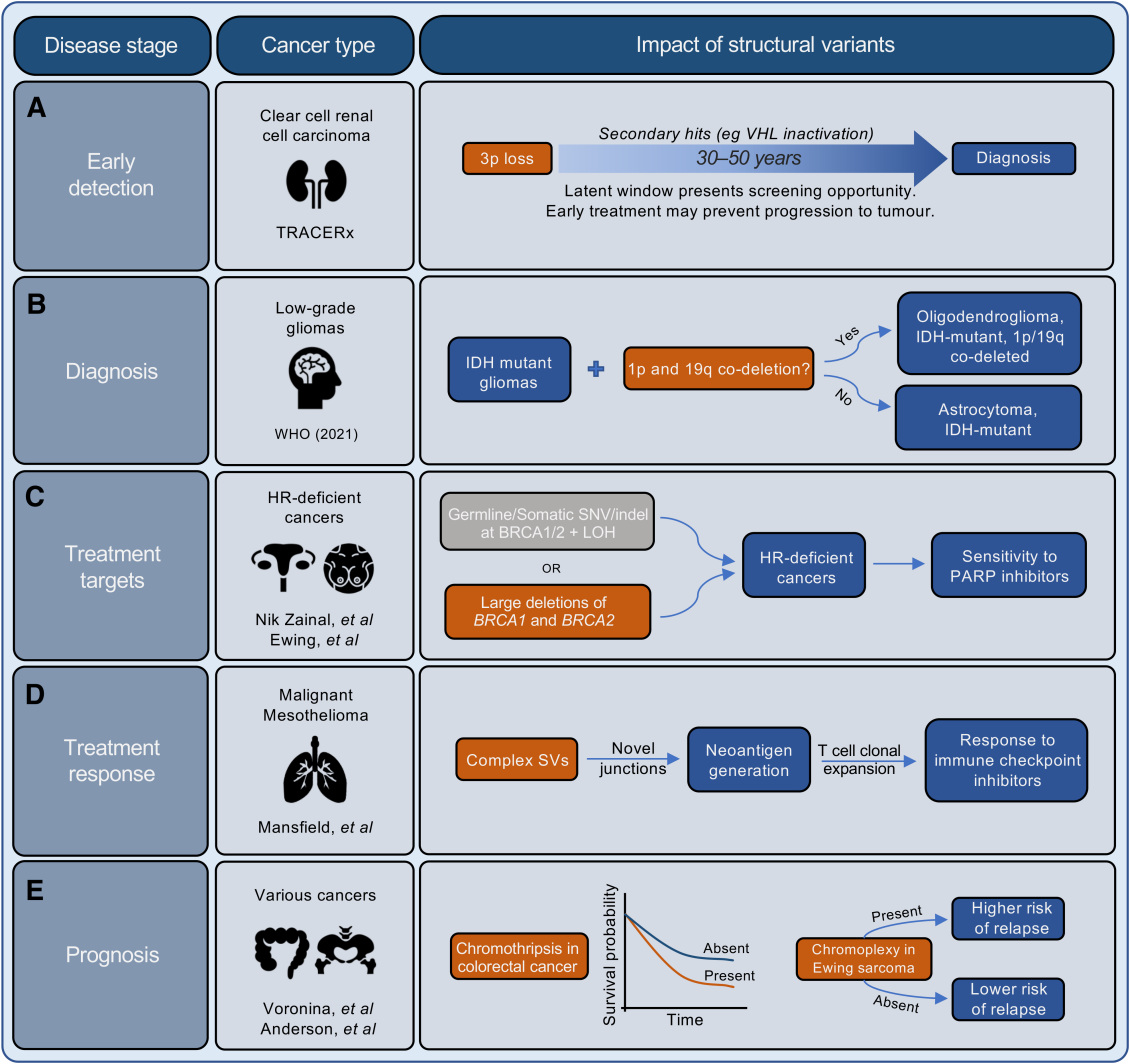
The clinical importance of structural variants (SVs) across all stages of the patient journey. SVs have significant potential in influencing the management of cancer patients throughout the disease trajectory. Some examples of the potential impact of SVs include: (A) Early detection: the TRACERx study on clear cell renal cell carcinoma [160] identified that 3p loss was frequently generated *via* chromothripsis, and that the loss could occur decades before diagnosis, suggesting the opportunity for early detection and intervention. (B) Diagnosis: the stratification of low‐grade gliomas (isocitrate dehydrogenase [IDH] mutant gliomas) has recently been updated by the WHO to include those with or without concurrent 1p and 19q co‐deletion [161]. (C) Treatment targets: tumours with large deletions of both *BRCA1* and *BRCA2* loci form a subset of HR‐deficient tumours that are exquisitely sensitive to PARP inhibitors [154, 155, 162]. (D) Treatment response: Mansfield *et al* [157, 158] demonstrated that malignant mesothelioma harbours neoantigens that are generated by complex SVs, and that their generation was associated with the clonal expansion of T cells, indicating that complex SV burden can be used to predict response to immune checkpoint inhibitors. (E) Prognosis: the presence of complex SVs are associated with poor prognosis. For example, patients with chromothripsis‐harbouring colorectal tumours have poorer survival rates compared to those without chromothripsis [163], and patients with Ewing sarcoma that contain chromoplexy have a higher risk of relapse following first‐line therapy [112].

Despite the many challenges in identifying clinically relevant SVs, recent findings uncover the translational potential of SVs as both causes (direct functional impact) and consequences (reflection of mutational processes) of cancer. While SV products such as gene fusions and amplifications are well known for their translational impacts (for example, as treatment targets [137, 159]), the potential clinical application of other SVs, particularly complex SVs, remain relatively unexplored. In the last decade, many studies have demonstrated proof‐of‐concept data highlighting the potential roles that SVs can play in every stage of cancer, from screening to treatment to prognostication (Figure 3). Premalignancy, driver SVs can exist decades before the onset of symptoms, generating opportunities for screening and early detection [160, 164, 165, 166, 167, 168]. The TRACERx group [160] showed that chromosome 3p loss is often the initiating driver in clear cell renal cell carcinoma and is predicted to arise 30–50 years before diagnosis (Figure 3A). In lung adenocarcinomas, driver fusion oncogenes, often derived from complex SVs, could occur decades before disease onset [164]. These findings suggest that in some cancer types a long latent window exists in which an effective screening strategy could be employed to allow earlier detection of cancer. However, it is important to consider when employing such a strategy that a single initiating driver may not be sufficient for cancer to develop and that for some individuals a necessary secondary event may never occur. SVs can also be used to predict progression to tumour from a premalignant state. In multiple myeloma, Oben *et al* [165] showed that the presence of myeloma‐defining genomic events, including chromothripsis in the rarely progressing precursor state, monoclonal gammopathy of undetermined significance (MGUS), can define a more progressive subset. In oesophageal cancer, complex SVs and CNAs are the most accurate predictors of malignant transformation from the Barrett's oesophagus state [166, 167, 168].

Following malignant transformation, the presence of specific SVs is pathognomonic of certain cancer types and can act as a diagnostic tool. For example, the prototypical oncoprotein *BCR‐ABL1* is diagnostic of chronic myeloid leukaemia [169]. Other diagnostic fusion genes include *PML‐RARA* in acute promyelocytic leukaemia [170], *TMPRSS2‐ERG* in prostate adenocarcinoma [171, 172, 173], and *EWSR1‐ETS* in Ewing sarcoma [112, 135, 174]. Other classes of SVs have been proposed as part of the established diagnostic criteria of certain cancers, including focal amplification or deletion (*EGFR* amplification in glioblastoma and *CDKN2A/B* deletion in IDH mutant astrocytoma [161]) and chromosomal gains and losses (chromosome 7 gain or 10 loss in glioblastoma, 1p and 19q losses in oligodendroglioma (Figure 3B) [175, 176]). Diagnosis based on these types of events is often indicative of their critical role in driving the development of the tumour, and as such many of the features used for diagnosis are effective targets for treatment.

Several oncogenic gene fusions are currently being used or explored as therapeutic targets in various cancer types [139]. The prototypical example of this is the use of imatinib mesylate to treat haematological cancers generating *BCR‐ABL* fusion oncoprotein [137, 169, 177]. More recently, targeting fusion transcripts has expanded into other cancer types. These include the application of inhibitors of *ALK*‐containing fusions in lung cancers [178] and anaplastic large‐cell lymphomas [169], *NTRK*‐containing fusions in various solid tumours [179, 180, 181], and *FCGR‐TACC* fusions in glioblastoma [182, 183] and other malignancies [184]. However, the utility of SVs as therapeutic targets is not limited to gene fusions. Significant progress has been made in some settings by targeting treatment based on oncogene amplification, including *ERBB2* in breast and ovarian cancers [159, 185], *EGFR* in breast, colorectal, and lung cancers [186, 187, 188], and *MYC* in neuroblastoma [189]. As evidence accumulates on the role of complex SVs, in particular of ecDNAs, on oncogene amplification, we would expect our grasp of the clinical utility of these events to strengthen. On the flip side, tumour suppressor loss is also an effective target for treatment. While restoring tumour suppressor function is challenging [190], the greatest clinical impact has come from exploiting vulnerabilities in cancer cells that lack functional tumour suppressor genes. This is the case when *BRCA1/2* function is lost in homology‐directed repair (HR) deficient breast, ovarian, prostate, and pancreatic cancers, conferring the tumours' sensitivity to PARP inhibition *via* synthetic lethality. *BRCA1/2* loss consists of a pathogenic SNV/indel in *BRCA1/2* in either the germline or somatic DNA followed by loss of the other allele *via* copy number neutral loss of heterozygosity or deletion [154, 162]. However, recent evidence has emerged from WGS that indicates that tumours with large heterozygous deletions spanning the length of both *BRCA1* and *BRCA2*, in the absence of SNVs/indels, are also more likely to be HR‐deficient and may also benefit from PARP inhibitors [155] (Figure 3C). This is an exciting exemplar, where the clinical impact of a known therapeutic target may be extended to a greater number of patients when we comprehensively account for the full structural complexity of tumour genomes.

In cases where SVs are not directly impacting tumour development or where the functional consequences of the SVs have not yet been determined, such as those SVs with breakpoints within a noncoding region, SVs can be used as biomarkers for treatment response. Tumours harbouring complex SVs such as chromothripsis and chromoplexy can generate various novel fusion junctions that result in the generation of neoantigens when expressed [112, 113, 135, 164, 191, 192]. For example, in malignant mesothelioma widespread complex SVs are predicted to generate neoantigens and correlate with clonal expansion of tumour infiltrating T lymphocytes [157, 193], suggesting that the presence of these complex SV events may be a useful biomarker for response to immune checkpoint inhibitors (Figure 3D) [194]. Further, tumours with ecDNAs may be more resistant to targeted oncogenic therapies due to their inherent plasticity and the significant contribution of ecDNAs to intratumoural heterogeneity [124]. In addition, overall genomic instability itself, rather than the presence of a particular SV, may be exploited as a treatment target [195]. A recent study suggested that drug inhibition against KIF18A, a key protein in the maintenance of spindle dynamics, resulted in antiproliferative effects specifically in tumours with increased chromosomal instability [196]. A further area of biomarker discovery lies in the application of mutational signatures [154, 162, 197]. These genome‐wide patterns of mutation have been extensively studied in the context of SNVs and are particularly useful for predicting exposure to mutational processes and DNA repair deficiencies. Although SV signatures are currently less well explored due to challenges in classifying SVs as a result of their diversity, patterns of tandem duplication and deletion are strongly associated with HR deficiency and can be used, together with the rest of the mutational landscape, as a proxy for treatment response [153, 154, 162]. We expect the clinical utility of SV signatures as biomarkers to expand as our understanding of the SV landscape develops and strategies for informative classification emerge.

Increased genomic instability as a result of ongoing chromosomal rearrangements has long been associated with poor prognosis in many cancer types [92, 198]. This association is likely driven by a greater propensity for treatment resistance as a result of the significant contribution of genomic instability to intratumoural heterogeneity [105]. Further, the association may also reflect analogous patterns of cell tolerance to extensive genomic rearrangements as to treatment onslaught. On the other hand, extreme levels of genomic instability, where most of the tumour genome is subject to chromosomal rearrangements and CNAs, are associated with better prognosis [199, 200]. In this instance, tumour cells with an overwhelming burden of genomic instability are on the brink of death, as so much of their genome is adversely altered, making successful replication unlikely. In addition, high levels of genomic instability render the tumour cells more immunogenic, resulting in a more efficient immune response and tumour clearance [201]. These seemingly conflicting findings suggest that a threshold of genomic instability may exist, such that tumour growth and adaptation are enhanced up to a certain level of instability, but that beyond this level, growth and survival may be compromised. This phenomenon suggests that tumour cells rely on an exquisite balance of genomic instability in ensuring their survival. More specifically, the impact of individual structural events on patient prognosis, other than where they are a target for treatment, is less clear. Complex SVs such as chromothripsis are associated with poor prognosis in multiple cancer subtypes (Figure 3E) [132, 163], including multiple myeloma [202], malignant melanoma [203], medulloblastoma [204], neuroblastoma [205], osteosarcoma [206], metastatic colorectal cancer [163, 207], and acute myeloid leukaemia [208, 209]. Similarly, ecDNAs are associated with poor outcome in aggressive cancers such as glioblastoma [210], neuroblastoma [211], medulloblastoma [70], and breast cancer [159]. Chromoplexy events in Ewing sarcoma portend the high risk of relapse [112] (Figure 3E). SVs are also a marker of late‐stage disease in malignant melanoma [212] and prostate cancer [213]. In general, those tumours that acquire a burst of mutations, perhaps through the generation of a complex SV, in a short period of time tend to proliferate rapidly and metastasize early to many differing sites, resulting in poorer clinical outcomes [92]. Overall, despite the significant challenges in identifying and interpreting the impact of SVs, their potential for substantial clinical impact, whether as diagnostic tools, therapeutic targets, or biomarkers for treatment response or patient prognosis, is clear.

## Conclusions and future perspectives

The SV landscape of a tumour genome in all its complexity holds tremendous potential for clinical impact at all stages of the patient's treatment journey, from early detection to predicting survival outcomes. However, if we are to capitalise on this opportunity to maximise the resultant benefit for patients, barriers with respect to the accurate detection and interpretation of the entire diverse SV landscape must be overcome. Our current understanding of this facet of tumour biology is based on insight gleaned from short‐read WGS from large tumour cohorts, which, due to technical limitations will necessarily underestimate the scale and complexity of these harder to study mutational events. Expanded use of long‐read sequencing technologies combined with improvements in analytical methods designed to encapsulate the full complexity of these events will address some of the current limitations. Nevertheless, challenges to interpreting the impact of these events will remain, to say nothing of the significant challenges in translating resultant insights to a clinical setting, which are the subject of many dedicated reviews [214, 215, 216].

Despite current limitations, existing studies provide an exciting glimpse into the emerging aspects of the genomic landscape of tumours, which represent opportunities to expand our understanding of tumour biology and provide novel avenues for translational exploitation in the future. Our rapidly developing appreciation for the impact that complex SVs have on tumour evolution and patient outcome is still in its infancy, but as the field develops we expect that these events along with the rest of the SV landscape will be crucial to unravelling the evolutionary trajectories that cells take on their routes to malignancy and metastases.

## Author contributions statement

Both authors contributed to all processes involved in writing this review and approved the final article.
